# X-Ray-Based 3D Histopathology of the Kidney Using Cryogenic Contrast-Enhanced MicroCT

**DOI:** 10.1155/2024/3924036

**Published:** 2024-04-09

**Authors:** Arne Maes, Onno Borgel, Clara Braconnier, Tim Balcaen, Martine Wevers, Rebecca Halbgebauer, Markus Huber-Lang, Greet Kerckhofs

**Affiliations:** ^1^Department of Materials Engineering, KU Leuven, Heverlee, Belgium; ^2^Biomechanics Lab, Institute of Mechanics, Materials and Civil Engineering, UCLouvain, Louvain-la-Neuve, Belgium; ^3^Pole of Morphology, Institute of Experimental and Clinical Research, UCLouvain, Brussels, Belgium; ^4^Institute of Clinical and Experimental Trauma-Immunology, University Hospital Ulm, Ulm, Germany; ^5^MolDesignS, Sustainable Chemistry for Metals and Molecules, Department of Chemistry, KU Leuven, Leuven, Belgium; ^6^Prometheus, Division for Skeletal Tissue Engineering, KU Leuven, Leuven, Belgium

## Abstract

The kidney's microstructure, which comprises a highly convoluted tubular and vascular network, can only be partially revealed using classical 2D histology. Considering that the kidney's microstructure is closely related to its function and is often affected by pathologies, there is a need for powerful and high-resolution 3D imaging techniques to visualize the microstructure. Here, we present how cryogenic contrast-enhanced microCT (cryo-CECT) allowed 3D visualization of glomeruli, tubuli, and vasculature. By comparing different contrast-enhancing staining agents and freezing protocols, we found that the preferred sample preparation protocol was the combination of staining with 1:2 hafnium(IV)-substituted Wells-Dawson polyoxometalate and freezing by submersion in isopentane at −78°C. This optimized protocol showed to be highly sensitive, allowing to detect small pathology-induced microstructural changes in a mouse model of mild trauma-related acute kidney injury after thorax trauma and hemorrhagic shock. In summary, we demonstrated that cryo-CECT is an effective 3D histopathological tool that allows to enhance our understanding of kidney tissue microstructure and their related function.

## 1. Introduction

Kidneys are vital organs with primary functions including the excretion of waste products from the blood, the regulation of the body's fluid balance, and the secretion of certain hormones [1]. To fulfill these functions, the kidney comprises a specialized and complex microstructure, containing glomeruli, diverse types of tubuli, and a highly branched vascular network [2]. Given the close links between microstructure and function, effective visualization methods are crucial to allow in-depth structural characterization of the kidney. Moreover, these methods could also provide important insights into the onset and progression of kidney diseases, as these often affect the kidney's microstructure [3–5].

Medical *in vivo* imaging techniques such as X-ray computed tomography (CT), magnetic resonance imaging (MRI), and ultrasound (US) are indispensable for clinical diagnostics of kidney diseases. Nonetheless, their spatial resolution is insufficient to fully capture the intricate microstructure of the kidney. As a result, various *ex vivo* imaging methods have been developed to histologically characterize the kidney, each possessing unique advantages and limitations. The current gold standard remains classical 2D histology, which involves embedding the sample and sectioning it into thin sections for subsequent staining with a colorimetric or fluorescent stain and analysis using a microscope (optical, fluorescent, or electron) [6, 7]. Classical 2D histology offers several advantages, including a broad range of available stains and a high discriminative power of tissue constituents (e.g., vascular network, glomeruli, and tubuli) down to the cellular level. Nevertheless, due to its inherent 2D nature, this imaging technique is unable to fully capture the intricate 3D microstructural organization of the kidney, such as the shape, size, and connectivity of tissue constituents. In addition, classical 2D histology is susceptible to sample distortion, folds, cracks, and tissue shrinkage, which can potentially change the native microstructure [8–10].

To overcome these limitations of classical 2D histology, different 3D *ex vivo* imaging techniques have been developed for the microstructural characterization of the kidney, including micro-MRI [11–13], optical coherence tomography (OCT) [14–16] and various microCT-based methods [11–16]. MicroCT has gained interest in recent years, as it offers both high spatial resolution (order of one micron) and the ability to image the entire organ. However, given the relatively low and similar X-ray attenuation of soft tissues, the image contrast between different types of soft tissues requires enhancement by either diffusing a contrast-enhancing staining agent (CESA) into the kidney (i.e. contrast-enhanced microCT; CECT) [17–19], exploiting the phase shift of the X-ray beam as it propagates through the sample (i.e. phase-contrast microCT; PCT) [20–23], or injecting a curable contrast agent into the vasculature of the kidney which forms a vascular cast [24–27]. CECT has emerged as a promising 3D imaging method for kidney since, unlike PCT, it does not require synchrotron-based imaging, and it is not limited to the visualization of the renal vasculature, which is the case for vascular casting. Nonetheless, it remains challenging to visualize certain tissue constituents, such as glomeruli or tubuli, without the use of destructive CESAs (or sample preparation steps) that induce tissue shrinkage or deformation. In this regard, cryogenic CECT (cryo-CECT), which was recently introduced by our group [28], was hypothesized to be a promising nondestructive visualization alternative for 3D histo(patho)logical imaging of the kidney.

The use of cryo-CECT could be of great benefit in the (pre-)clinical kidney research of acute kidney injury (AKI) after trauma. AKI is a common posttraumatic complication, especially after hemorrhagic shock, and associated with a high morbidity and mortality rate [29, 30]. The causes of trauma-related AKI (TRAKI) are complex and include ischaemic, direct trauma-related, septic, and toxic pathomechanisms [31]. As a consequence, the microcirculation is disturbed post trauma [32], which in turn, can lead to apoptosis and necrosis of renal tissue [33]. However, so far, studies addressing microstructural changes of the kidneys by indirect trauma are mostly limited to classical 2D histology, which is less sensitive in detecting very localized alterations and less accurate in quantifying 3D structural parameters. Compared to other kidney disease models, such as classical ischemia-reperfusion injury (IRI), sepsis-induced AKI, or autoimmune-associated AKI, the morphological changes in the TRAKI model are more subtle. Therefore, it is well suited for challenging the sensitivity of cryo-CECT. This study is aimed at demonstrating the potential of cryo-CECT to visualize the microstructure of the kidney. To achieve this, we evaluated the effectiveness of three different CESAs for both CECT and cryo-CECT imaging of the murine kidney. Moreover, different freezing rates were compared to identify the optimal freezing protocol that enables microstructural visualization while preserving tissue integrity. Using cryo-CECT, we were able to identify, segment, and quantify structural parameters of various tissue constituents from the kidney's microstructure, such as individual glomeruli, tubuli, and vascular structures. Finally, we also evaluated cryo-CECT in a mouse model of mild TRAKI by meticulously modelling a typical trauma situation to demonstrate its potential in (pre-)clinical application for X-ray-based 3D histopathology.

## 2. Materials and Methods

### 2.1. Animals

For the technique optimization experiments, 9 kidneys, each originating from a different mouse (strain B6C3Fe) of 17 weeks old, were recuperated from experimental material that was kindly donated by the IREC Morphology Laboratory (UCLouvain) (ethical approval number 2021/UCL/MD/047).

The animal experiment on mild TRAKI was performed in accordance with the ARRIVE guidelines after approval of the Animal Welfare Officer of the University of Ulm and the Regierungspräsidium Tübingen (approval number: 1409). In brief, four to five C57BL/6J mice per group (24 h control *n* = 5, 24 h trauma *n* = 4, and 5 d trauma *n* = 5), aged 10-12 weeks, were investigated under sufficient anaesthesia. Blunt thoracic trauma was induced by a defined blast wave and hemorrhagic shock modeled by a pressure-controlled mean arterial pressure (MAP) of 30 ± 5 mmHg for 60 min as described elsewhere [34, 35]. The drawn blood was anticoagulated with 2 IE heparin. After the shock phase, animals were retransfused with the collected blood within 15 min. The animals were then continuously observed for one hour and given slowly 2 × 500 *μ*L jonosteril via the inserted femoral artery catheter. After the procedure, the artery line was removed, the skin was sutured, and animals had unrestricted access to food and water. At 4, 24 or 120 h, respectively, animals were sacrificed and the bladder was punctured for urine collection. The left kidney was removed after ligation of the vessels.

Following dissection, all kidneys were fixed using 4% formaldehyde (FA) dissolved in PBS for 24 h at 4°C. Afterwards, samples were stored in fresh phosphate-buffered saline (PBS) at 4°C until staining with a CESA.

### 2.2. Contrast-Enhancing Staining Agents (CESAs)

Three different CESA solutions were prepared: two based on Lugol's iodine (I_2_KI; “Lugol PBS” and “B-Lugol”) and one based on 1:2 hafnium(IV)-substituted Wells-Dawson polyoxometalate (Hf-WD POM; K_16_[Hf(*α*_2_-P_2_W_17_O_61_)_2_]•19H_2_O). Lugol's iodine was prepared at a physiological osmolality (312 mOsm/kg) by dissolving 12.948 g KI and 6.474 g I_2_ in 500 mL Milli-Q water. This solution was then diluted 1:1 (volume ratio) with either isotonic PBS (10 mM, pH = 7.4; “Lugol PBS”) or the stronger Sörensen buffer (266 mM, pH = 7.2) following the protocol of buffered Lugol (“B-Lugol”) as described by Dawood et al. [36]. The Hf-WD POM solution was prepared by dissolving 35 mg/mL of Hf-WD POM powder in PBS. All samples were immersed in 5 mL of staining solution (solution-to-sample volume ratio of 20:1) for 10 days while placed on a horizontal shaker plate at room temperature.

### 2.3. Freezing Protocols

After removing kidneys from the staining solution, they were wrapped in two layers of Parafilm™ to avoid direct contact with the isopentane. For sample freezing, the wrapped kidneys were submerged for 1 minute in isopentane that was cooled down to either −20°C, −78°C or −160°C. To achieve the first freezing protocol, a bottle of isopentane was placed in the −20°C freezer overnight. During this freezing protocol, the bottle was placed in a styrofoam box and surrounded by ice packs (−20°C) to stabilize the isopentane's temperature. For the freezing protocol with isopentane at −78°C, a bottle of isopentane was precooled overnight in the −80°C freezer and placed in a styrofoam box while surrounded by dry ice (−78°C). For the freezing protocol with isopentane at −160°C, a metal beaker filled with isopentane was partially submerged in liquid nitrogen (-196°C) causing the bottom portion of the isopentane to solidify (*T*_m_ = −160°C), while the top portion remained liquid and reached thermal equilibrium at −160°C. For all freezing protocols, the temperature of the isopentane was inspected using a low-temperature thermometer (Traceable™ LN2 Excursion-Trac™, Traceable™ Products, Webster, Texas, USA).

### 2.4. MicroCT Image Acquisition and Reconstruction

Overview datasets (entire kidney), central zoom datasets (central portion of the kidney), and upper pole datasets (trauma and corresponding sham animals) were acquired using a Phoenix Nanotom M (GE Measurement and Control Solutions, Germany) equipped with a 180 kV/15 W energy X-ray tube source. A diamond-coated tungsten target was used for all acquisitions. Image acquisition parameters are summarized in Table 1. For cryo-CECT imaging, the in-house developed *in situ* cryostage, as introduced in our recent study [28], was installed and cooled down to an ambient temperature of -35°C. MicroCT datasets were reconstructed using the Datos|x software (GE Measurement and Control Solutions) with the beam hardening correction (value: 8), inline median filter, and ROI-CT filter active. Reconstructed slices were exported as XY slices (.tiff). An in-house developed MATLAB script was used to convert the 16-bit slices (.tiff) to 8-bit slices (.bmp), while simultaneously windowing the histogram range to the dynamic range of the dataset [37]. For the overview datasets, two reference material beads (alumina (Al_2_O_3_) and polypropylene (PP)) were included to calculate normalized grey values allowing relative grey value comparison.

Cortical zoom datasets at high spatial resolution (voxel size of 1.5) were acquired using a TESCAN UniTOM HR system (TESCAN XRE, Belgium) equipped with a 160 kV/25 W energy X-ray tube source. The detector had a field of view of 2916 × 2280 pixels with a 50 *μ*m pixel pitch. Image reconstruction was performed using the TESCAN Panthera software. Image acquisition parameters are summarized in Table 1.

### 2.5. MicroCT Image Analysis

Based on the two reference material beads (Al_2_O_3_ and PP), normalized grey values of the different kidney layers were calculated. First, the average grey value of these regions, and of the reference materials, was measured by drawing an oval-shaped region of interest (ROI) in the different kidney layers and calculating the average grey value within the ROIs. This was performed on a coronal microCT slice through the middle of the kidney using ImageJ software (U. S. National Institutes of Health, Bethesda, Maryland, USA). The normalized grey values (NGVs) of the different types of renal tissue were then calculated using

(1) $$\begin{array}{c} \text{NG}{\text{V}}_{\text{Tissue}}=\frac{\text{G}{\text{V}}_{\text{Tissue}}-\text{G}{\text{V}}_{\text{PP}}}{\text{G}{\text{V}}_{\text{A}{\text{l}}_{2}{\text{O}}_{3}}-\text{G}{\text{V}}_{\text{PP}}}. \end{array}$$

**Figure 1:**
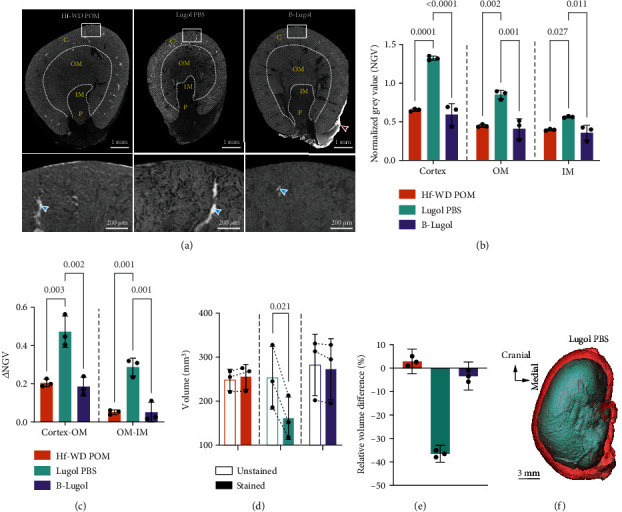
Comparison of three different CESA solutions for conventional CECT imaging of kidney. (a) Transverse CECT slices through the middle of the kidney showing the different layers of the kidney including the cortex (C), outer medulla (OM), inner medulla (IM), and papillae (P). A zoom image of the cortex (white rectangle) is shown below, with the blue arrows indicating stained blood inside the vasculature. The highly attenuating adipose tissue at the hilum caused by the B-Lugol staining is indicated by the red arrow. Image histograms were windowed based on their dynamic range. Hence, images are not displayed as normalized grey values in-between different datasets. (b, c) Bar graphs showing the (b) normalized grey values (NGVs) of the kidney layers and the (c) difference in NGVs between neighboring layers for the different staining solutions. (d–f) The kidney volume changes due to the staining process. Bar graphs showing the (d) volume of the kidneys prior to and after staining and the (e) relative volume changes, for the different staining groups. Volume rendering of the same kidney prior to (red) and after staining (blue) with Lugol PBS (f). The bars in the bar graphs represent the mean, with individual data points also indicated by the dots. The error bars indicate the standard deviation in (b)–(d) and the 95% confidence interval in (e). For each kidney layer, unpaired one-way analysis of variance, followed by a two-sided Tukey's test, was conducted in (b) and (c) to compare the different staining solutions. Two-sided paired *t*-tests were conducted to compare groups (before and after staining) in the bar graphs of (d). Significant *p* values (*p* < 0.05) have been indicated in the bar graphs.

**Figure 2:**
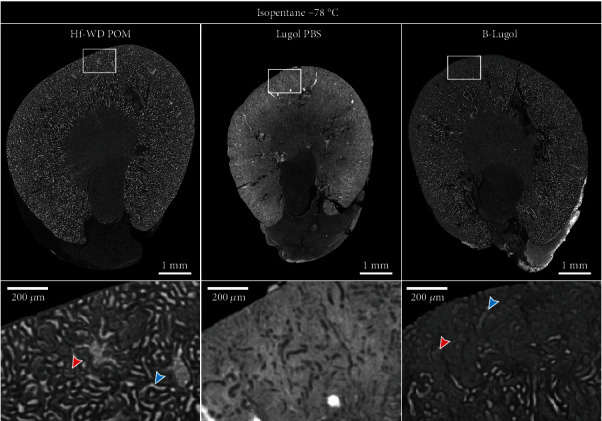
Evaluating the potential of the different CESA solutions for cryo-CECT (isopentane −78°C). Transverse cryo-CECT slices of kidneys that were stained by either one of the CESA solutions (Hf-WD POM, Lugol PBS, or B-Lugol) and, subsequently, frozen by submersion in isopentane at −78°C. A magnification (white rectangle) of the cortex is shown below. Hf-WD POM and B-Lugol allowed the visualization of glomeruli (red arrow) and tubuli (blue arrow). Image histograms were windowed based on their dynamic range. Hence, images are not displayed as normalized grey values in-between different datasets.

3D visualization and analysis of the datasets were performed using the Avizo 2022.1 software (Thermo Fisher Scientific, Bordeaux, France). From the overview datasets, kidneys were semiautomatically segmented from the background and parafilm using the magic wand tool (grey value-based region growing), allowing to quantify the sample volume prior to and after staining. Relative volume changes were then calculated using equation (2). From the central zoom datasets (cryo-CECT), Bowman's capsules of glomeruli in the cortex were manually segmented from the surrounding tissue. The glomeruli inside the capsules were then semiautomatically segmented based on grey value using the magic wand tool. The distance-to-capsule of a glomerulus was manually measured on transverse microCT slices. Various 3D structural parameters were determined for each glomerulus using label analysis, including volume, sphericity (equation (3)), and equivalent diameter (equation (4)). From the cortical zoom dataset, the renal arterial vasculature and individual partial nephrons were semiautomatically segmented using the *magic wand tool* in Avizo 2022.1 software. This tool performs a region growing operation in 3D by selecting all the voxels which (i) fall inside the user-defined range of grey values and (ii) are spatially connected to the seed point selected by the user. Different microCT datasets, as well as classical 2D histological sections, of the same sample (Figures 1 and 2) were automatically registered and aligned using Avizo 2022.1 software.

For the TRAKI kidneys, a papillary tubule analysis was performed by first extracting a subvolume (600 *μ*m × 600 *μ*m × 600 *μ*m) in the papillary region for each animal. The papillary lumen was then segmented automatically using the watershedding algorithm in Avizo. A thickness analysis was performed in CTAn software (v. 1.18.9.0; Bruker MicroCT, Kontich, Belgium), and the absolute volume was determined by label analysis using Avizo. Automated segmentation of the medullary tubuli from the surrounding tissue was not feasible due to the more heterogenous grey values of the lumens and interstitium, compared to the papillae. Therefore, the diameter of the lumen (*D*_lumen_) and the lumen with interstitium (*D*_lumen+int_) were assessed manually in transverse sections through the medulla of the upper pole of the kidney. In multiple sections located in this region, we measured 20 tubules per animal, which were oriented orthogonally to the transverse plane. Per tubule, one 2D measurement of lumen and interstitium was performed. The width of the interstitium (*W*_int_) was then calculated using equation (5) (Supplementary Figure 1).

(2) $$\begin{array}{c} \Delta V_{\text{rel}}=\frac{V_{\text{stained}}-V_{\text{unstained}}}{V_{\text{unstained}}}, \end{array}$$

(3) $$\begin{array}{c} \text{Sphericity}=\frac{\pi^{1/3}\;{\left(6\times \text{volume}\right)}^{2/3}}{\text{surface}}, \end{array}$$

(4) $$\begin{array}{c} \text{Equivalent diameter}=\sqrt[{3}]{\frac{6\times \text{volume}}{\pi },} \end{array}$$

(5) $$\begin{array}{c} W_{\mathrm{int}}=\frac{D_{\text{lumen}+\mathrm{int}}-D_{\text{lumen}}}{2}. \end{array}$$

### 2.6. Classical 2D Histological Assessment

After microCT imaging, samples were rinsed in PBS for 2 days before being embedded in paraffin using a Vacuum Infiltration Tissue Processor (Tissue-Tek VIP, Sakura Finetek, Netherlands). Transversal sections of 5 *μ*m thick were made using a microtome and stained with hematoxylin and eosin (H&E) for comparison with cryo-CECT. Images of the histological sections were obtained using a SCN400 Slide Scanner (Leica Microsystems, Germany).

### 2.7. Neutrophil Gelatinase-Associated Lipocalin (NGAL) Measurements

To detect kidney damage, urinary neutrophil gelatinase-associated lipocalin (NGAL) levels were measured using a commercially available ELISA kit (R&D, Minneapolis, USA) according to the manufacturer's instructions with a Sunrise™ absorbance microplate reader (Tecan, Männedorf, Switzerland). All measurements were made in duplicate.

### 2.8. Statistical Analysis

The software GraphPad Prism (GraphPad Software, California, USA) was used for statistical analysis. *p* values below 0.05 were considered significant and are indicated in the bar graphs. The mean value of the different samples is indicated by the height of the bars. Individual data points for each sample are indicated in the bar graphs. Error bars represent, unless specified otherwise in the figure legend, the standard deviation. The type of statistical test performed is described in the figure legends.

## 3. Results

### 3.1. Hf-WD POM Allows to Visualize the Kidney's Microstructure Using (Cryo-)CECT While Avoiding Sample Shrinkage

Before performing cryo-CECT imaging, three different staining solutions were compared in terms of their CECT visualization potential of the kidney's microstructure and their effect on sample volume (Figure 1): Hf-WD POM, Lugol PBS, and B-Lugol [36]. All three staining solutions allowed to differentiate between the main layers of the kidney: the cortex, the outer medulla (OM), the inner medulla (IM), and the papillae (Figure 1(a)). Whereas Hf-WD POM and B-Lugol provided a similar visualization of the kidney's microstructure, the Lugol PBS staining resulted in a noticeably higher image contrast between the different regions. In addition, the Lugol PBS most clearly revealed the tubular microstructure. Interestingly, the staining behavior of the B-Lugol solution was altered compared to the conventional Lugol PBS solution, which is in contradiction to what was published before [36]. In addition to the lower image contrast between the different regions compared to the Lugol PBS, the B-Lugol also resulted in a strong staining of the adipose tissue at the hilum (red arrow in Figure 1(a)), which was not observed with the other staining solutions. To better visualize the staining differences between the Lugol PBS and B-Lugol, normalized CECT slices are shown in Supplementary Figure 2.

Normalized grey values (NGVs) of the different kidney layers were calculated to compare the different staining solutions quantitatively (Figures 1(b) and 1(c)). The NGVs represent the degree of X-ray attenuation and, in the case of the B-Lugol and Lugol PBS, the local iodine concentration. For all regions measured (cortex, OM, and IM), the Lugol PBS resulted in significantly higher NGVs compared to other staining groups (Figure 1(b)). This indicates that the Lugol PBS caused an overall higher iodine uptake compared to the B-Lugol. Similar NGVs were observed for the Hf-WD POM and B-Lugol staining. Next, we calculated the difference in NGVs (*Δ*NGV) of neighboring regions since this indicates the ease of differentiating between them (Figure 1(c)). Again, the Lugol PBS staining resulted in significantly higher *Δ*NGV compared to the Hf-WD POM or B-Lugol, reflecting the higher image contrast between neighboring regions in Figure 1(a). The NGV analysis also quantitatively confirmed the significant effect of the buffer solution on the staining behavior of Lugol's iodine.

Pair-wise comparisons of the same kidney prior to and after staining revealed only for the Lugol PBS group a significant decrease in volume (−36.5%) (Figures 1(d) and 1(e)). The 3D volume renderings of the kidney before and after staining with the Lugol PBS provide a visual demonstration of the substantial sample shrinkage caused by the staining process (Figure 1(f)). This tissue shrinkage induced by the Lugol PBS could lead to inaccurate quantitative analyses of the renal microstructure. It is worth noting that sample freezing, using isopentane at -78°C, did not induce significant volume changes (Supplementary Figure 3), which was also recently observed by Pestiaux et al. for murine hearts [38].

Next, the cryo-CECT potential of the different staining solutions was evaluated by freezing the stained kidneys by submersion in isopentane at −78°C and imaging them in their frozen state (Figure 2). The freezing-induced visualization enhancement of the kidney's microstructure was least pronounced for the Lugol PBS staining, with only minor visual differences between the CECT and cryo-CECT images. Because of this observation, combined with the substantial sample shrinkage, the Lugol PBS was excluded from further analysis. Applying cryo-CECT on the kidneys stained with the Hf-WD POM or B-Lugol considerably enhanced the visualization of microstructural details, such as individual glomeruli, tubuli, and vasculature. Compared to the B-Lugol, Hf-WD POM provided slightly more detail and image contrast using cryo-CECT. We qualitatively validated the visualization of the various microstructural constituents by cryo-CECT on the Hf-WD POM-stained kidneys by comparing registered slices to classical 2D histological sections of the same kidney (Supplementary Figure 4). In addition, it is worth mentioning that solely freezing, without prior CESA staining, also enables the visualization of certain microstructural constituents albeit at a lower image contrast (Supplementary Figure 5).

### 3.2. Submersion in Isopentane at −78°C Is the Preferred Freezing Protocol for Hf-WD POM-Stained Kidneys

**Figure 3:**
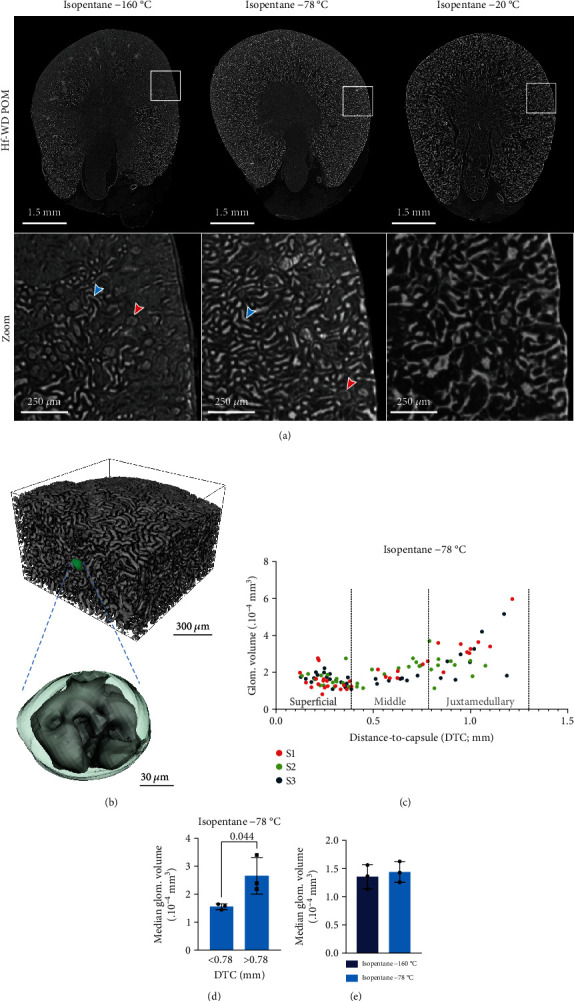
Optimization of the freezing rate for cryo-CECT and quantitative structural characterization of the glomeruli. (a) Transverse cryo-CECT slices of Hf-WD POM-stained kidneys that were frozen by submersion in isopentane at either −160°C, −78°C, or −20°C. A zoom image of the cortex (white rectangle) is shown below. Glomeruli and tubuli are indicated by the red and blue arrows, respectively. Image histograms were windowed based on their dynamic range. Hence, images are not displayed as normalized grey values in-between different datasets. (b) Typical volume rendering of the cortical kidney microstructure (Hf-WD POM and isopentane −78°C). The Bowman's capsule of a glomerulus is rendered in green. A magnification of the Bowman's capsule (green transparent) and the glomerulus (grey) is shown. (c) Scatter plot showing the relationship between glomerular volume and the distance-to-capsule of each glomerulus measured for all samples (S1, S2, and S3) that were frozen using isopentane −78°C. The cortex is divided in three regions (superficial, middle, and juxtamedullary) according to Zhai et al. [39]. (d) Bar graph comparing the median glomerular volume between the juxtamedullary cortex (DTC > 0.78 mm) on the one hand and superficial and middle cortex (DTC < 0.78 mm) on the other hand. (e) Bar graph comparing the median glomerular volume (15 glomeruli per kidney; either superficial or middle) between the two freezing rates. Two-sided unpaired *t*-tests were performed. Significant *p* values (*p* < 0.05) have been indicated in the bar graphs.

Given the clearest visualization by Hf-WD POM using cryo-CECT (Figure 2), we used Hf-WD POM for further optimization of the cryo-CECT imaging and image analysis. As shown in our recent study introducing cryo-CECT [28], the freezing rate is a crucial parameter to obtain optimal visualization of the tissue constituents while also preserving the tissue's microstructure. By submerging the Hf-WD POM-stained kidneys in isopentane at either −160°C, −78°C, or −20°C, we were able to evaluate the effect of the freezing rate on both visualization and tissue integrity of the kidney (Figure 3). The slowest freezing rate (isopentane −20°C) had a considerable effect on the microstructure, with tubuli and glomeruli being compressed by the excessive growth of ice crystals. As a result, this freezing rate made it impossible to distinguish individual tubuli or glomeruli (Figure 3(a)). The highest freezing rate (isopentane −160°C) allowed the visualization of glomeruli and tubuli in the cortex (red and blue arrow in Figure 3(a), respectively). However, some cortical regions near the capsule were lacking microstructural details resulting in an inhomogeneous visualization of the tubuli and glomeruli. The intermediate freezing rate (isopentane −78°C) resulted qualitatively in the best balance between preserving tissue integrity and a more uniform visualization of the kidney's microstructure.

Interestingly, we observed a trend towards larger glomeruli as the distance of the glomerulus to the capsule increased (Figures 3(c) and 3(d)). Following the study of Zhai et al. [39], the cortex was divided into three regions based on the distance-to-capsule: superficial (0–0.39 mm), middle (0.39–0.78 mm), and juxtamedullary (0.78–1.3 mm). Especially in the juxtamedullary cortex, the glomeruli were on average larger and showed a higher variation in size compared to the glomeruli in the superficial and middle cortex (Figure 3(d)).

Next, we quantified the microstructural effect of increasing the isopentane's temperature from −160°C to −78°C, thus lowering the freezing rate. To this end, the median glomerular volume was measured based on 15 individual glomeruli per kidney, located in the superficial or middle regions of the cortex (Figure 3(e)). Similar values for the median glomerular volume indicate that, compared to freezing with isopentane at −160°C, lowering the freezing rate to isopentane −78°C does not substantially alter the microstructure. Also for the other structural parameters of either the glomeruli or Bowman's capsules, no significant differences were observed (Supplementary Figure 6). Taking this into account, together with the more homogeneous visualization of the tissue constituents and the decreased risk of freezing cracks, isopentane −78°C was selected as the preferred freezing protocol for cryo-CECT imaging of kidney.

### 3.3. Hf-WD POM-Based Cryo-CECT (Isopentane at −78°C) Allows High-Resolution Visualization of Individual Nephrons

**Figure 4:**
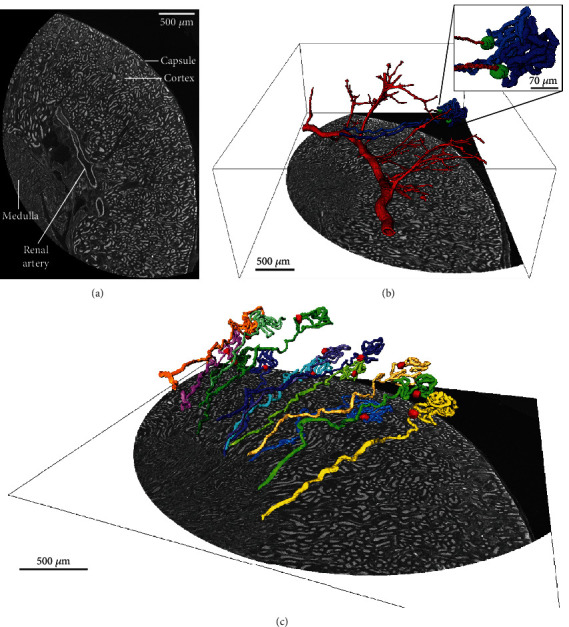
Segmentation of individual partial nephrons based on the cortical zoom dataset. (a) Transverse cryo-CECT image of the cortical zoom dataset (voxel size: 1.5 *μ*m) showing the capsule, the cortex, part of the medulla, and the renal artery. (b) Volume rendering of the renal artery (red) branching out into the cortex, connecting to the glomeruli (green) of two nephrons (blue). (c) Volume renderings of several partial nephrons showing the glomerulus (red), proximal convoluted tubule, and the thick descending limb of loop of Henle.

From the cortical zoom dataset, we were able to segment the renal arterial vasculature, as well as parts of individual nephrons containing the glomerulus, proximal convoluted tubule, and the thick descending limb of the loop of Henle (Figures 4(a) and 4(b)). The volume rendering also shows the branching of the renal artery into the afferent arterioles, which eventually connect to the glomeruli (Supplementary Video 1). At this high spatial resolution (voxel size of 1.5 *μ*m), the field of view of the microCT system was too limited to image the entire volume of the kidney at once. However, if required, multiple image datasets can be acquired and merged to achieve full-organ imaging.

The segmentation of the nephrons was performed semiautomatically, which allowed rapid segmentation of multiple nephrons in the kidney's cortex without the need for manual drawing (Figure 4(c) and Supplementary Video 2). Nevertheless, the current semiautomatic segmentation workflow was not able to track the tubule where it transitions from thick descending limb into thin descending limb in the inner medulla due to changes in grey value and structure. As a result, parts of the loop of Henle and the distal convoluted tubule were not included in the segmentation in this study.

### 3.4. Cryo-CECT Can Detect Small Microstructural Changes in a Mild TRAKI Model

We applied cryo-CECT imaging to the kidneys of a trauma-related AKI (TRAKI) mouse model to evaluate the potential of cryo-CECT in detecting pathological changes to the kidney's microstructure. Representative cryo-CECT slices of control and trauma groups are shown in Supplementary Figure 7. The AKI biomarker NGAL was significantly elevated in urine in the 24 h trauma group, indicating manifestation of AKI. Concerning its dynamic, it was already recovered in the 5-day trauma group (Supplementary Figure 8).

**Figure 5:**
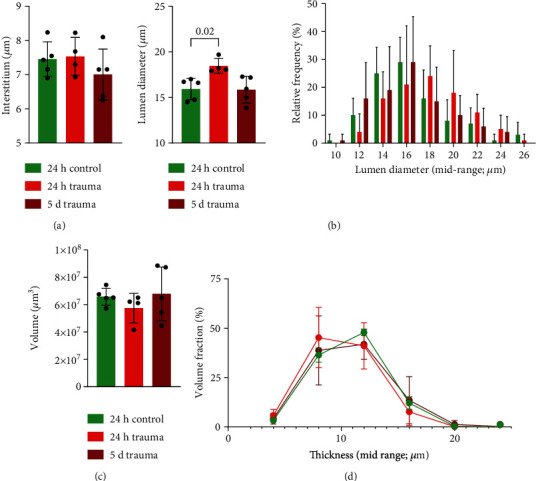
3D microstructural analysis of the medullary and papillary tubuli system in mice after trauma. Medullary tubule analysis with bar graphs showing (a) interstitial width as well as (b) lumen diameter and its relative frequency distribution. Papillary tubule analysis including the (c) total volume of papillary lumen per group and the (d) histogram of the tubuli thickness for the different experimental groups. One-way analysis of variance, followed by a two-sided Tukey's test, was conducted. Significant *p* values are indicated above the graphs (*p* < 0.05).

Since trauma can affect the tubular system of the kidney indirectly and directly, we assessed the medullary and papillary tubuli system using cryo-CECT (Figure 5). First, the medullary tubule system was examined by measuring the diameter of the lumen and the lumen with interstitium (Supplementary figure 1). The interstitial width in the 24-hour and 5-day trauma groups did not differ significantly from the 24-hour control group (Figure 5(a)). However, the diameter of the lumen significantly increased in the 24-hour trauma group compared to the 24-hour control group, indicating luminal congestion rather than interstitial edema. This increase was restored in the 5-day trauma group (Figure 5(b)).

The papillary tubule system was assessed by segmentation of the tubule lumens, followed by a quantification of the volume, as well as the thickness distribution, of the lumens. We found a slight absolute volume reduction in the 24 h trauma group compared to the 24 h control group, indicating some stenosis of the papillary lumen (Figure 5(c)). This was not evident in the 5-day trauma group, but the standard deviation of the data was also higher in the 5-day trauma group, compared to the 24 h control group, showing an inconsistent recovery. The thickness analysis showed that this volume reduction in the 24 h trauma group was particularly evident for tubuli with thicknesses ranging from 10 *μ*m to 16 *μ*m (Figure 5(d)).

**Figure 6:**
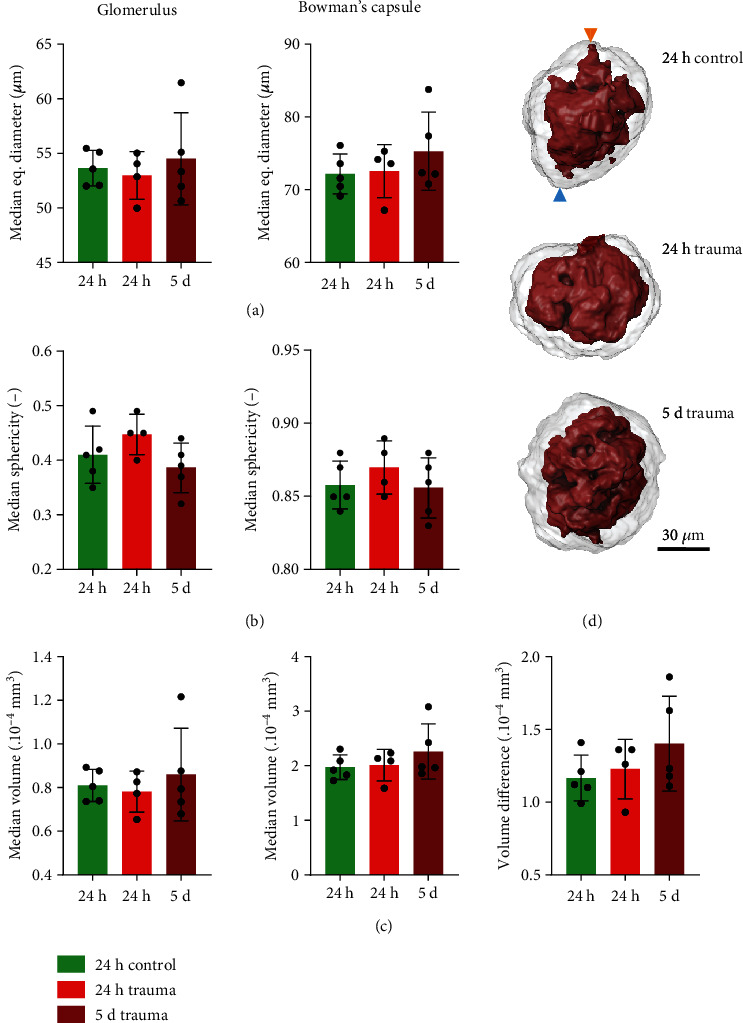
3D microstructural analysis of glomeruli in mouse kidney after trauma. (a) Median equivalent diameter, (b) median sphericity, and (c) median volume for both glomerulus and Bowman's capsule, as well as the (c) volume difference between Bowman's capsule and glomerulus. (d) Typical volume renderings of the Bowman‘s capsule with corresponding glomerulus, for the different experimental groups (orange arrow: vascular pole, green arrow: urinary pole). One-way analysis of variance, followed by a two-sided Tukey's test, was conducted. No significant *p* values (*p* < 0.05) were observed.

To evaluate the structural effect of the trauma on the glomeruli, we segmented 15 individual glomeruli and the corresponding Bowman's capsule for each kidney, located in the superficial or middle region of the cortex, from the cryo-CECT data. Cryo-CECT allowed to differentiate and quantify these two structures in 3D. Comparing the different 3D structural parameters, we observed a slight trend towards larger median equivalent diameter and median volume for both Bowman's capsule and glomerulus in the 5-day trauma group (Figures 6(a) and 6(c)), whereas the sphericity of the Bowman's capsule and the glomerulus was the largest in the 24 h trauma group compared to the other groups (Figure 6(b)). Additionally, we observed a trend towards a larger difference in volume between the Bowman's capsule and glomerulus volume in the 5-day trauma group compared to the 24 h control group (Figure 6(c)), implying that the volume of the Bowman's capsule increased more than the volume of the glomerulus. Furthermore, the spread of data points for the volume difference was wider in the 5-day trauma group than in the 24 h control group.

## 4. Discussion

Cryo-CECT has been recently introduced by our group as 3D histo(patho)logical method for soft biological tissues, demonstrating its potential for skeletal muscle, heart, and tendon tissue [28]. In this study, we have further extended the application range of cryo-CECT to include kidney tissue and have applied it to a TRAKI mouse model to show its potential for X-ray-based 3D histopathology. To this end, three different staining solutions were compared in terms of their staining-induced sample shrinkage and visualization potential, both for CECT and cryo-CECT.

Changing the buffer of Lugol's iodine solution from regular PBS (10 mM) to the stronger Sörensen buffer (133 mM) almost completely prevented tissue shrinkage, confirming the findings of Dawood et al. [36]. However, we also observed significant changes in staining behavior between the Lugol PBS and B-Lugol. The overall increase of normalized grey values could potentially be linked to the sample shrinkage induced by the Lugol PBS as for the same uptake of iodine, the local iodine concentration would be higher. However, this does not explain the increased affinity of the B-Lugol towards the adipose tissue. It is tempting to speculate that the buffer capacity of the solution influences the reactivity and distribution of the different iodine species during the staining process and, hence, also the staining behavior of Lugol's iodine staining solution. For instance, it has already been shown that the rate of iodination of tyrosine and histidine is dependent on the pH and concentration of iodide [40–42].

For all staining solutions, cryo-CECT enhanced the visualization of the kidney's microstructure in comparison to conventional CECT. This enhancement was predominantly pronounced for the kidneys stained with Hf-WD POM and B-Lugol. For the Lugol PBS-stained kidneys, cryo-CECT only showed a minor improvement in visualization of the microstructure. This observation is in accordance with our previous study, in which cryo-CECT only had a minor effect on the Lugol PBS-stained muscle samples [28]. Presumably, this is linked to sample dehydration induced by the Lugol PBS staining, limiting the potential of the freezing step to enhance microstructural visualization.

For the Hf-WD POM-stained kidneys, we found that the isopentane −78° C freezing protocol resulted in the optimal balance between uniform visualization of the microstructural details, while preserving tissue integrity. However, slight compression of the cortical tubuli was observed occasionally, resulting in a closing of the lumen (Figure 3). It is unclear at the moment whether this closing of the lumen is caused by the freezing step, prior sample preparation steps or the filling of the lumen with CESA. Nevertheless, the true optimal isopentane's temperature may lie between −160°C and −78°C. However, stabilizing the temperature of the isopentane between −160°C and −78°C without specialized equipment is challenging.

Cryo-CECT allowed not only the visualization but also the 3D quantitative structural characterization of microstructural constituents, such as individual glomeruli. The analysis showed a significant increase in glomerular volume between cortical and juxtamedullary glomeruli in healthy kidneys. This observation has already been made by others [43–46]. However, there is still debate whether this change in size is inherent or induced by various factors such as aging, obesity, or glomerulosclerosis. As such, cryo-CECT could be a valuable method to gain insight into this phenomenon, as it allows accurate quantification of the glomerular volume for various conditions. Compared to conventional 2D histological methods, cryo-CECT is superior in terms of accuracy for glomerular size evaluation since it does not depend on the sectioning depth or orientation through the glomerulus. Instead, the glomerular volume is directly measured based on the 3D dataset.

From the cortical zoom cryo-CECT datasets at higher spatial resolution (voxel size of 1.5 *μ*m), we were able to semiautomatically segment the arterial vasculature and parts of individual nephrons (Figure 4). This allowed us to visualize the transition of the afferent arterioles into the glomeruli. The 3D connectivity of these constituents, as well as the intricate trajectory of tubuli, is structural information which is beyond the reach of classical 2D histology. This information, however, would be vital to better understand the microstructural effect of various disorders [47, 48] and pathologies [49, 50] affecting the tubular architecture (e.g., cystic kidney disease [50]). As such, the 3D visualization of the kidney's microstructural constituents could provide valuable insights into the structure and function of both healthy and pathological kidneys.

Severe trauma and hemorrhage are known to cause various changes in the kidney's structure, including villi degradation, tubular necrosis, and disruption of the blood-urine barrier [31]. However, corresponding studies on the structural changes of trauma in mouse kidneys are mostly limited to classical 2D histology. Here, cryo-CECT could add complementary 3D microstructural information concerning TRAKI-induced alterations. Previously, we have demonstrated that cryo-CECT is very sensitive in detecting minimal pathology-related structural changes [28]. Now, we have shown that this also applies in a model of mild TRAKI, revealing a significant enlargement of the medullary tubular lumen and in some cases an increased volume difference between the Bowman's capsule and the glomerulus. Furthermore, segmentation of the glomeruli, separated from the Bowman's capsule, allowed a differentiated view of pathological changes in the filtration units and thereby could provide additional information about glomerular pathologic changes such as edema, glomerular fibrosis, or vasoconstriction. We have also established techniques to measure pathomorphological changes in the papillary and medullary tubule system. By analyzing the lumen and interstitium separately, we were able to differentiate between luminal dilatation and processes that enlarge the interstitial space, such as edema or infiltration. We have demonstrated that TRAKI also results in a broader distribution of microstructural parameters such as glomerular and Bowman's capsule median equivalent diameter and volume, as well as papillary lumen volume during the later time course. Furthermore, we observed an early widening of the lumen of the medullary tubules. The outer medulla in particular is at risk of hypoxic damage [32]. Therefore, it is tempting to speculate that the disturbed homeostasis seen in the microstructural changes and debris deposition could lead to a combination of congestion and edema resulting in the detected wider lumen [31]. Overall, these microstructural changes could sufficiently explain the development of posttraumatic renal dysfunction [34]. In the future, the cryo-CECT-generated images can advantageously be matched with identical sections obtained by immunohistochemistry and in their combination enable mechanistic structure-function analysis. For instance, GATA3 and podoplanin immunohistochemical staining was still successful on kidneys that underwent cryo-CECT (Supplementary Figure 9). In addition, several (immuno)histochemical stainings following (cryo-)CECT based on Hf-WD POM have been described in literature, including CD31 immunostain [28, 51], Verhoeff's Van Gieson stain [52], Sirius red stain [28], and Masson's trichrome [28]. Now that the proof of concept has been established, other murine kidney disease models with more severe/localized morphologic changes, such as IRI, sepsis-induced, or autoimmune-associated AKI, could be studied using the cryo-CECT technique.

Although this study was limited to the analysis of murine kidneys, cryo-CECT could also be applied in the future for 3D histo(patho)logy on human kidney tissue, either from a biopsy or a cadaveric donor. Kidney biopsies are frequently collected in hospitals to help diagnose kidney disease or assess the severity of kidney damage [53]. Using a special needle, a small piece of tissue is taken from the kidney for histological assessment by a pathologist. In this regard, cryo-CECT could provide a valuable 3D histo(patho)logical imaging technique, complementary to classical 2D histology, to better diagnose or assess kidney damage.

While cryo-CECT shows great promise as a novel method for 3D histology of kidney, it is not without its limitations. Tissue cracking can occur at high freezing rates (isopentane −160°C), leading to potential sample damage. Furthermore, validation with classical 2D histology was only performed qualitatively. Given the substantial tissue shrinkage during sample preparation for classical 2D histology (Supplementary Figure 10), a quantitative comparison between cryo-CECT and classical 2D histology could lead to erroneous conclusions. Similar observations regarding tissue shrinkage due to paraffin embedding have been made for murine hearts [28, 38] and for porcine aortic wall tissue [52]. Also, the current semiautomatic segmentation method was not able to keep track of the tubule when it transitioned from thick descending limb to thin descending limb in the inner medulla. This prevented the segmentation of the entire nephron including the loop of Henle and the distal convoluted tubule. More advanced segmentation approaches that are, for instance, based on artificial intelligence could aid in more effective segmentation of individual nephrons. Finally, despite the recent technological advancements in the field of microCT imaging, classical 2D histology remains superior in terms of in-plane spatial resolution.

## 5. Conclusions

Cryo-CECT represents a promising approach to X-ray-based 3D histo(patho)logy of the kidney with the potential to enhance our understanding of kidney tissue microstructure and function. We found that a combination of Hf-WD POM staining and freezing by submersion in isopentane at −78°C was superior to other tested methods in visualizing various tissue constituents while minimizing alterations to the microstructure. Furthermore, these constituents, including glomeruli, vasculature, and tubuli, could be segmented and structurally quantified in full 3D based on the cryo-CECT data. This technique is also capable of detecting subtle, minor pathological differences in mice with a mild trauma-related acute kidney injury. Because of its 3D nature, it is possible to differentiate between different regions of the kidney without destroying the material for later classical 2D histological analysis. This may be valuable for future structure-function research in other kidney diseases, especially for those with heterogeneous pathological changes or with early but minimal microstructural changes.

## Figures and Tables

**Table 1:** MicroCT acquisition parameters.

Acquisition parameters	Overview	Central zoom	Cortical zoom	Upper pole (TRAKI)
MicroCT system	GE Nanotom M	GE Nanotom M	UniTOM HR	GE Nanotom M
Voxel size (*μ*m)	8	3	1.5	2
Tube voltage (kV)	60^∗^/80	80	80	90
Tube power (W)	—	—	3	—
Tube current (*μ*A)	250	155	—	90
Nr. of projections	1200	2400	2800	2100
Exposure time (ms)	500	500	500	750
Average	1	2	4	2
Skip	—	1	—	1
Acquisition time (min)	10	60	105	80
Source-detector-distance (mm)	500	333	495	450
Source-sample-distance (mm)	40	10	15	9
CECT or cryo-CECT	Both	Both	Cryo-CECT	Cryo-CECT
Grey value normalization	Yes	No	No	No

^∗^Corresponds to tube voltage (60 kV) used for the image acquisition of unstained kidney.

## Data Availability

The microCT datasets generated and/or analyzed during the current study are not publicly available due to their considerable size but are available from the corresponding authors on request. The in-house developed MATLAB script to convert the reconstructed 16-bit slices (.tiff) to 8-bit slices (.bmp or .jpg), while simultaneously windowing the histogram range to the dynamic range of the dataset, is available at https://github.com/contrast-team/histogram-windowing (doi:10.5281/zenodo.7034265).

## Supplementary Materials

Supplementary Materials Supplementary Figure 1: medullary tubule analysis. Supplementary Figure 2: normalized overview CECT slices of kidneys stained with the Lugol PBS or B-Lugol. Supplementary Figure 3: effect of freezing using isopentane at -78°C on the volume of the kidneys. Supplementary Figure 4: qualitative validation of cryo-CECT imaging by comparison to classical 2D histology. Supplementary Figure 5: the enhanced visualization of freezing on an unstained kidney. Supplementary Figure 6: quantitative results of the structural characterization of the glomeruli and Bowman's capsules. Supplementary Figure 7: representative cryo-CECT slices for control and trauma groups in the TRAKI model. Supplementary Figure 8: NGAL in urine after trauma. Supplementary Figure 9: immunohistochemistry performed on kidneys that underwent cryo-CECT imaging (Hf-WD POM staining and freezing using isopentane at –78°C). Supplementary Figure 10: the tissue shrinkage induced by dehydration and paraffin embedding as preparation for classical 2D histology. Supplementary Video 1: 3D rendering of the kidney's vasculature and two individual nephrons. Supplementary Video 2: 3D rendering of several nephrons.

## References

[1] Kaur S., Kaur M., Singh N. P., Prabhakar H., Gupta N. (2020). Normal physiology of renal system. *Brain and Kidney Crosstalk*.

[2] Paulsen F., Waschke J. (2013). Kidney and adrenal gland. *Sobotta Atlas of Human Anatomy, Vol. 2*.

[3] Gunawardena S., Dayaratne M., Wijesinghe H., Wijewickrama E. (2021). A systematic review of renal pathology in chronic kidney disease of uncertain etiology. *Kidney International Reports*.

[4] Liu H., Feng J., Tang L. (2022). Early renal structural changes and potential biomarkers in diabetic nephropathy. *Frontiers in Physiology*.

[5] Hommos M. S., Glassock R. J., Rule A. D. (2017). Structural and functional changes in human kidneys with healthy aging. *Journal of the American Society of Nephrology*.

[6] Mescher L. A. (2016). Junqueira’s Basic Histology. *Text and Atlas*.

[7] Alturkistani H. A., Tashkandi F. M., Mohammedsaleh Z. M. (2015). Histological stains: a literature review and case study. *Global Journal of Health Science*.

[8] Hillman H. (2000). Limitations of clinical and biological histology. *Medical Hypotheses*.

[9] Taqi S. A., Sami S. A., Sami L. B., Zaki S. A. (2018). A review of artifacts in histopathology. *Journal of Oral and Maxillofacial Pathology: JOMFP*.

[10] Bindhu P. R., Krishnapillai R., Thomas P., Jayanthi P. (2013). Facts in artifacts. *J Oral Maxillofac Pathol*.

[11] Bennett K. M., Zhou H., Sumner J. P. (2008). MRI of the basement membrane using charged nanoparticles as contrast agents. *Magnetic Resonance in Medicine*.

[12] Xie L., Bennett K. M., Liu C., Johnson G. A., Zhang J. L., Lee V. S. (2016). MRI tools for assessment of microstructure and nephron function of the kidney. *American Journal of Physiology-Renal Physiology*.

[13] Beeman S. C., Zhang M., Gubhaju L. (2011). Measuring glomerular number and size in perfused kidneys using MRI. *American Journal of Physiology. Renal Physiology*.

[14] Chen Y., Andrews P. M., Aguirre A. D., Schmitt J. M., Fujimoto J. G. (2007). High-resolution three-dimensional optical coherence tomography imaging of kidney microanatomy ex vivo. *Journal of Biomedical Optics*.

[15] Konkel B., Lavin C., Wu T. T. (2019). Fully automated analysis of OCT imaging of human kidneys for prediction of post-transplant function. *Biomedical Optics Express*.

[16] Andrews P. M., Chen Y., Onozato M. L. (2008). High-resolution optical coherence tomography imaging of the living kidney. *Laboratory Investigation*.

[17] Missbach-Guentner J., Pinkert-Leetsch D., Dullin C. (2018). 3D virtual histology of murine kidneys -high resolution visualization of pathological alterations by micro computed tomography. *Scientific Reports*.

[18] Busse M., Mueller M., Kimm M. A. (2018). Three-dimensional virtual histology enabled through cytoplasm-specific X-ray stain for microscopic and nanoscopic computed tomography. *Proceedings of the National Academy of Sciences of the United States of America*.

[19] de Bournonville S., Vangrunderbeeck S., Ly H. G. T. (2020). Exploring polyoxometalates as non-destructive staining agents for contrast-enhanced microfocus computed tomography of biological tissues. *Acta Biomaterialia*.

[20] Shirai R., Kunii T., Yoneyama A. (2014). Enhanced renal image contrast by ethanol fixation in phase-contrast X-ray computed tomography. *Journal of Synchrotron Radiation*.

[21] Zdora M.-C., Thibault P., Kuo W. (2020). X-ray phase tomography with near-field speckles for three-dimensional virtual histology. *Optica*.

[22] Walsh C. L., Tafforeau P., Wagner W. L. (2021). Imaging intact human organs with local resolution of cellular structures using hierarchical phase-contrast tomography. *Nature Methods*.

[23] Wu J., Takeda T., Thet Lwin T. (2009). Imaging renal structures by X-ray phase-contrast microtomography. *Kidney International*.

[24] Xu R. D., Franchi F., Miller B. (2013). Polycystic kidneys have decreased vascular density: a micro-CT study. *Microcirculation*.

[25] Ehling J., Babickova J., Gremse F. (2016). Quantitative micro-computed tomography imaging of vascular dysfunction in progressive kidney diseases. *Journal of the American Society of Nephrology*.

[26] Hlushchuk R., Zubler C., Barre S. (2018). Cutting-edge microangio-CT: new dimensions in vascular imaging and kidney morphometry. *American Journal of Physiology-Renal Physiology*.

[27] Xie L., Koukos G., Barck K. (2019). Micro-CT imaging and structural analysis of glomeruli in a model of adriamycin-induced nephropathy. *American Journal of Physiology-Renal Physiology*.

[28] Maes A., Pestiaux C., Marino A. (2022). Cryogenic contrast-enhanced microCT enables nondestructive 3D quantitative histopathology of soft biological tissues. *Nature Communications*.

[29] Søvik S., Isachsen M. S., Nordhuus K. M. (2019). Acute kidney injury in trauma patients admitted to the ICU: a systematic review and meta-analysis. *Intensive Care Medicine*.

[30] Nasu T., Ueda K., Kawashima S. (2021). Prehospital blood pressure and lactate are early predictors of acute kidney injury after trauma. *Journal of Surgical Research*.

[31] Messerer D. A. C., Halbgebauer R., Nilsson B., Pavenstädt H., Radermacher P., Huber-Lang M. (2021). Immunopathophysiology of trauma-related acute kidney injury. *Nature Reviews Nephrology*.

[32] Wu C. Y., Chan K. C., Cheng Y. J., Yeh Y. C., Chien C. T. (2015). Effects of different types of fluid resuscitation for hemorrhagic shock on splanchnic organ microcirculation and renal reactive oxygen species formation. *Critical Care*.

[33] Cannon J. W. (2018). Hemorrhagic shock. *New England Journal of Medicine*.

[34] Denk S., Weckbach S., Eisele P. (2018). Role of hemorrhagic shock in experimental polytrauma. *Shock*.

[35] Wrba L., Ohmann J. J., Eisele P. (2019). Remote intestinal injury early after experimental polytrauma and hemorrhagic shock. *Shock*.

[36] Dawood Y., Hagoort J., Siadari B. A. (2021). Reducing soft-tissue shrinkage artefacts caused by staining with Lugol’s solution. *Scientific Reports*.

[37] Maes A. (2020). *Cryogenic Contrast-Enhanced MicroCT Enables Nondestructive 3D Quantitative Histopathology of Soft Biological Tissues*.

[38] Pestiaux C., Marino A., Simal L., Horman S., Capoulade R., Kerckhofs G. (2024). X-ray-based 3D histology of murine hearts using contrast-enhanced microfocus computed tomography (CECT) and cryo-CECT. *Hearts*.

[39] Zhai X.-Y., Thomsen J. S., Birn H., Kristoffersen I. B., Andreasen A., Christensen E. I. (2006). Three-dimensional reconstruction of the mouse nephron. *Journal of the American Society of Nephrology*.

[40] Dunford H. B., Adeniran A. J. (1988). The mechanism of the nonenzymatic iodination of tyrosine by molecular iodine. *Biochemistry and Cell Biology*.

[41] Dunford H. B., Ralston I. M. (1983). On the mechanism of iodination of tyrosine. *Biochemical and Biophysical Research Communications*.

[42] Wolff J., Covelli I. (1969). Factors in the iodination of histidine in proteins. *European Journal of Biochemistry*.

[43] Marumoto H., Tsuboi N., D’Agati V. D. (2021). Total nephron number and single-nephron parameters in patients with IgA nephropathy. *Kidney*.

[44] Newbold K. M., Sandison A., Howie A. J. (1992). Comparison of size of juxtamedullary and outer cortical glomeruli in normal adult kidney. *Virchows Archiv. A, Pathological Anatomy and Histopathology*.

[45] Skov K., Nyengaard J. R., Patwardan A., Mulvany M. J. (1999). Large juxtamedullary glomeruli and afferent arterioles in healthy primates. *Kidney International*.

[46] Artacho-Perula E., Roldan-Villalobos R., Salcedo-Leal I., Vaamonde-Lemos R. (1993). Stereological estimates of volume-weighted mean glomerular volume in streptozotocin-diabetic rats. *Laboratory Investigation; a Journal of Technical Methods and Pathology*.

[47] Walsh S. B., Unwin R. J. (2012). Renal tubular disorders. *Clinical Medicine*.

[48] Galarreta C. I., Forbes M. S., Thornhill B. A. (2015). The swan-neck lesion: proximal tubular adaptation to oxidative stress in nephropathic cystinosis. *American Journal of Physiology. Renal Physiology*.

[49] Schelling J. R. (2016). Tubular atrophy in the pathogenesis of chronic kidney disease progression. *Pediatric Nephrology*.

[50] Blanc T., Goudin N., Zaidan M. (2021). Three-Dimensional Architecture of Nephrons in the Normal and Cystic Kidney. *Kidney International*.

[51] Kerckhofs G., Stegen S., van Gastel N. (2018). Simultaneous three-dimensional visualization of mineralized and soft skeletal tissues by a novel microCT contrast agent with polyoxometalate structure. *Biomaterials*.

[52] Leyssens L., Balcaen T., Pétré M. (2023). Non-destructive 3D characterization of the blood vessel wall microstructure in different species and blood vessel types using contrast-enhanced microCT and comparison with synthetic vascular grafts. *Acta Biomaterialia*.

[53] Agarwal S. K., Sethi S., Dinda A. K. (2013). Basics of kidney biopsy: a nephrologist’s perspective. *Indian Journal of Nephrology*.

