# Supplementary material for: X-Ray-Based 3D Histopathology of the Kidney Using Cryogenic Contrast-Enhanced MicroCT
Source: Int J Biomed Imaging. 2024 Apr 9;2024:3924036. doi: 10.1155/2024/3924036 (PMC11022514; doi:10.1155/2024/3924036)
Supplement: Supplementary Materials — Supplementary Figure 1: medullary tubule analysis. Supplementary Figure 2: normalized overview CECT slices of kidneys stained with the Lugol PBS or B-Lugol. Supplementary Figure 3: effect of freezing using isopentane at -78°C on the volume of the kidneys. Supplementary Figure 4: qualitative validation of cryo-CECT imaging by comparison to classical 2D histology. Supplementary Figure 5: the enhanced visualization of freezing on an unstained kidney. Supplementary Figure 6: quantitative results of the structural characterization of the glomeruli and Bowman's capsules. Supplementary Figure 7: representative cryo-CECT slices for control and trauma groups in the TRAKI model. Supplementary Figure 8: NGAL in urine after trauma. Supplementary Figure 9: immunohistochemistry performed on kidneys that underwent cryo-CECT imaging (Hf-WD POM staining and freezing using isopentane at –78°C). Supplementary Figure 10: the tissue shrinkage induced by dehydration and paraffin embedding as preparation for classical 2D histology. Supplementary Video 1: 3D rendering of the kidney's vasculature and two individual nephrons. Supplementary Video 2: 3D rendering of several nephrons. [file 3924036.f1.zip › Revised Supplementary Figures and Videos - Kidney cryo-CECT (1).docx]

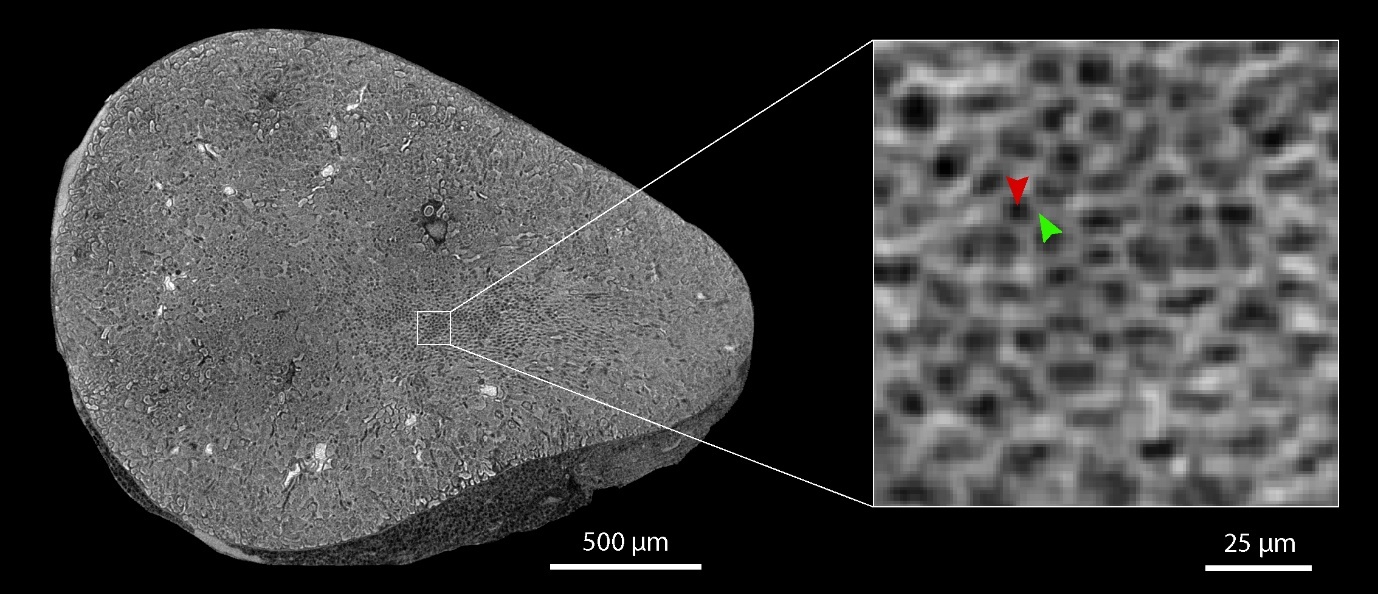


**Supplementary Fig. 1|Medullary tubule analysis**. Transverse cryo-CECT slice through the upper pole with the visualization of the measurement process of the medullary tubuli (green arrow: interstitium; red arrow: lumen). Tubules that were cut orthogonally in the transverse plane were measured.


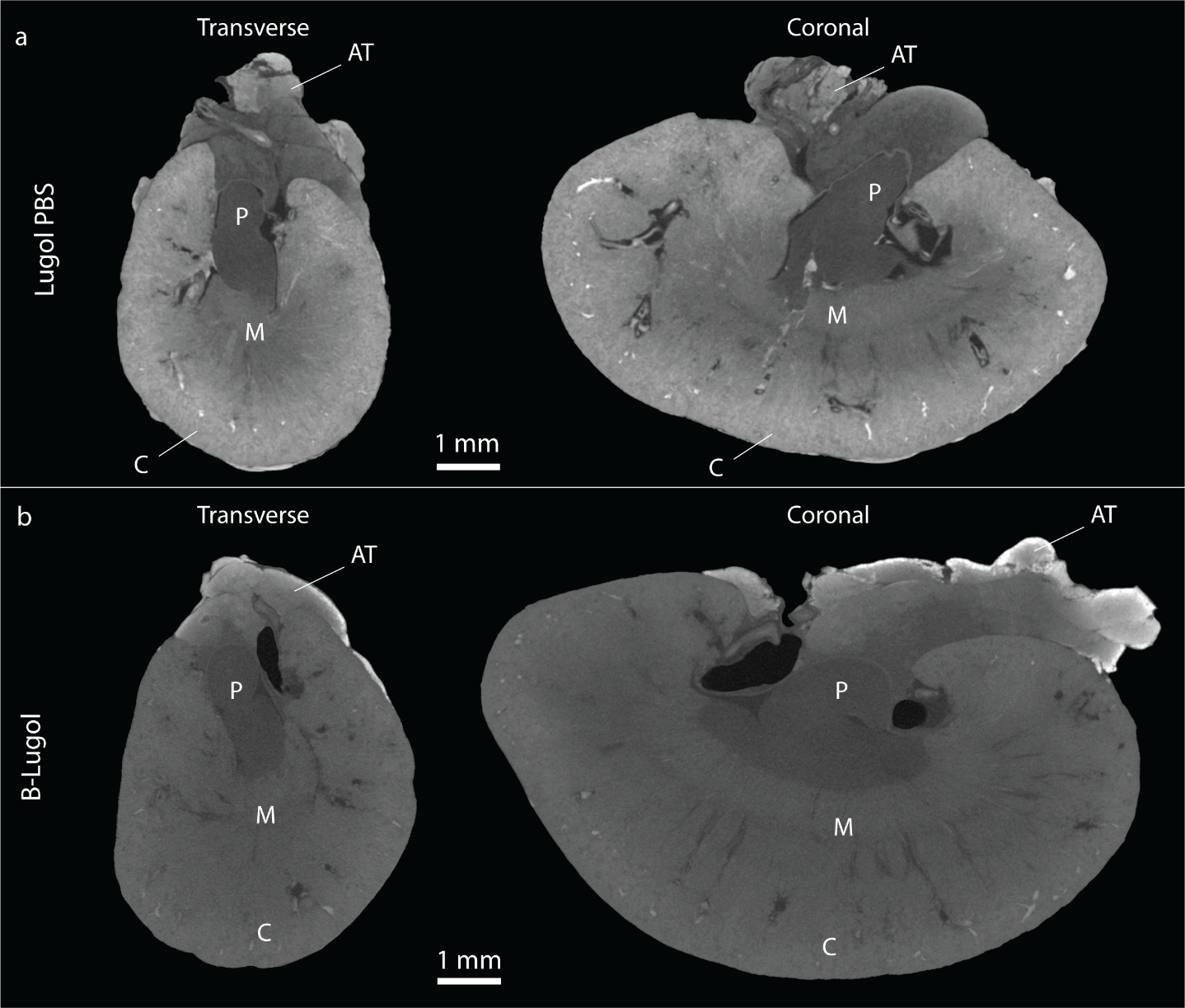


**Supplementary Fig. 2| Normalized overview CECT slices of kidneys stained with Lugol PBS or B-Lugol.** CECT cross-sectional images (left = transverse and right = coronal) of a murine kidney stained with either Lugol PBS (**b**) or B-Lugol (**c**). Grey values are normalized between (a) and (b) and can, therefore, be used to directly compare iodine content in the different kidney structures between Lugol PBS and B-Lugol. Different kidney structures are indicated: AT = adipose tissue, P = papilla, M = medulla, C = cortex.


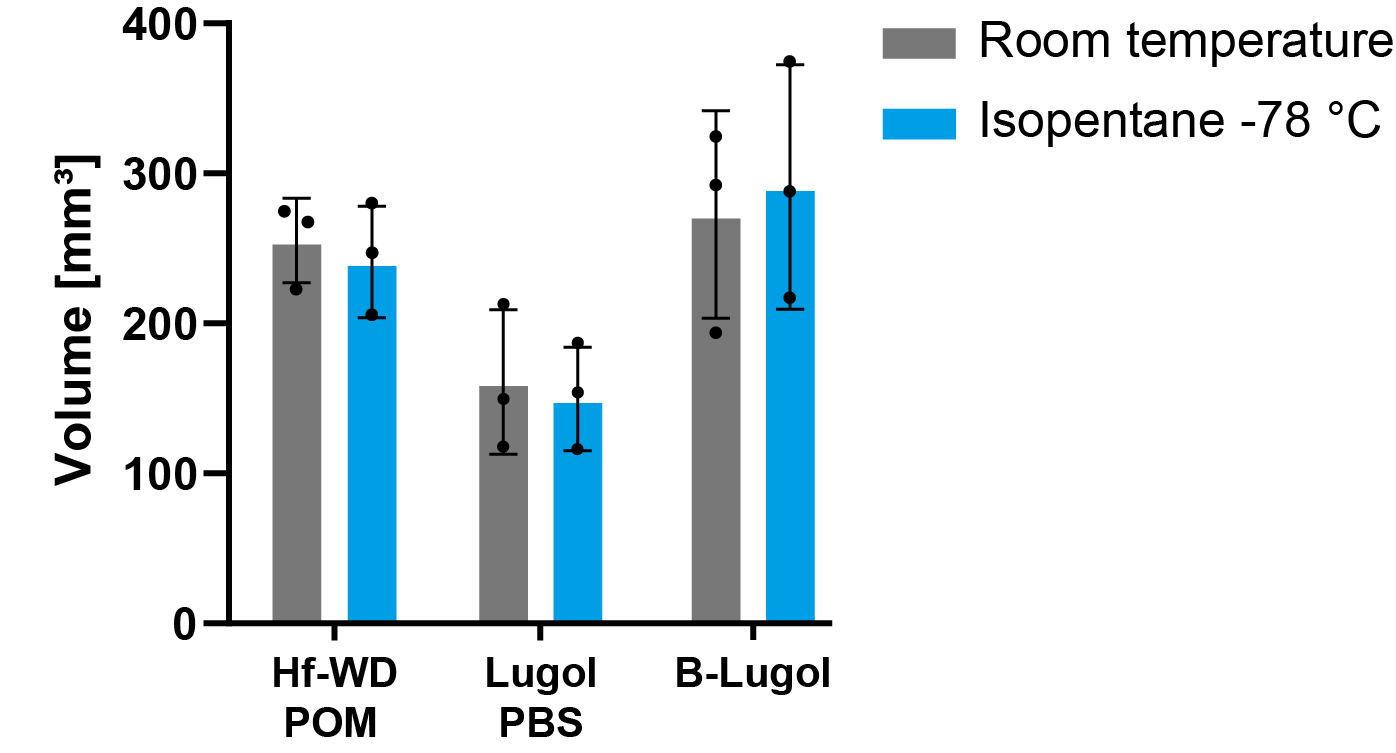


**Supplementary Fig. 3| Effect of freezing using isopentane at -78 °C on the volume of the kidneys.** Bar graphs showing the comparison of the volume of stained kidneys at room temperature and frozen using isopentane at -78 °C. The bars in the bar graphs represent the mean, with individual data points (n = 3 for each condition) indicated by the dots. The error bars indicate the standard deviation. Two-sided unpaired t-testing resulted in no significant differences (p > 0.05).


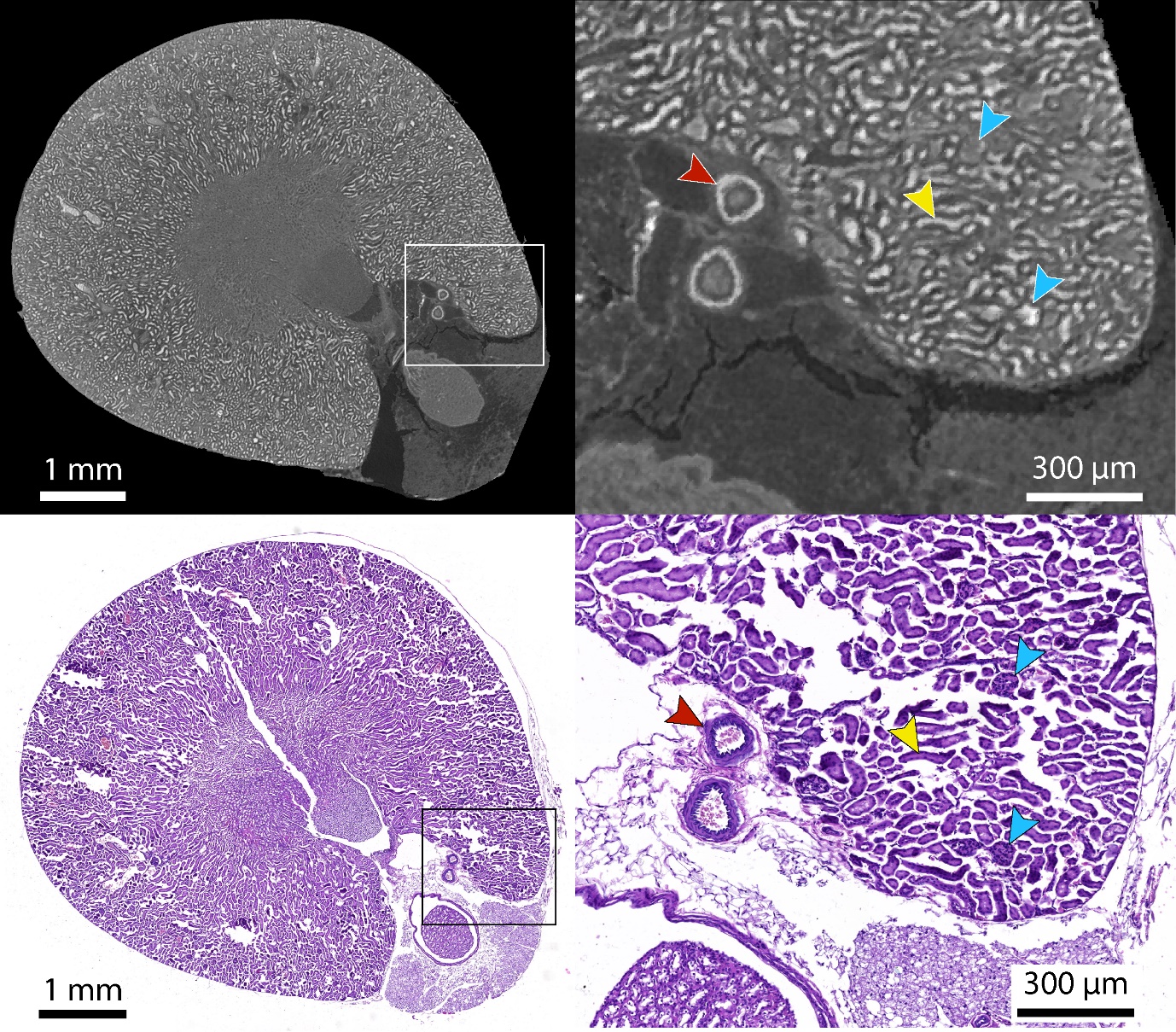


**Supplementary Fig. 4| Qualitative validation of cryo-CECT imaging by comparison to classical 2D histology.** Matching slices of the same kidney obtained by cryo-CECT (top; Hf-WD POM and isopentane -78 °C freezing) and classical 2D histology (bottom; H&E staining). Several microstructural constituents have been indicated on the zoom inset: artery (red arrow), tubulus (yellow arrow) and glomeruli (blue arrows). Several processing-related cracks and tearing can be observed in the 2D histological sections.


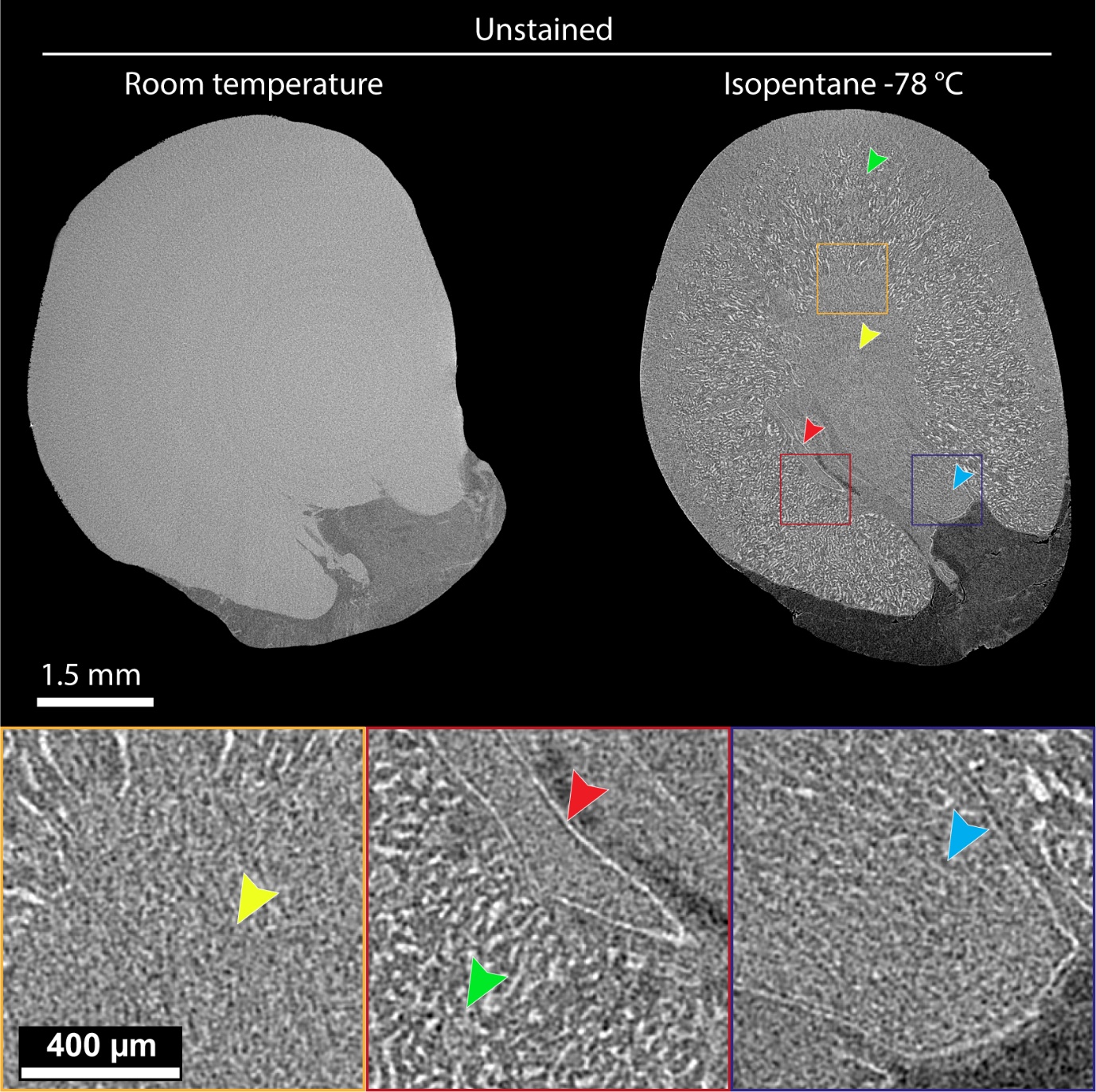


**Supplementary Fig. 5| The enhanced visualization of freezing on an unstained kidney.** Transverse slices of an unstained kidney at room temperature (left) and frozen using isopentane at -78 °C (right). At room temperature, the microstructure of the kidney cannot be discerned, besides the adipose tissue at the hilum. Solely freezing enables the visualization of the cortex (green arrow), medulla (yellow arrow), a renal artery (red arrow), and the papillae (blue arrow). Image contrast is, however, limited due to the lack of prior staining with a CESA. Magnifications of the colored rectangles are shown below. Image histograms were windowed based on their dynamic range. Hence, images are not displayed as normalized grey values in-between different datasets.


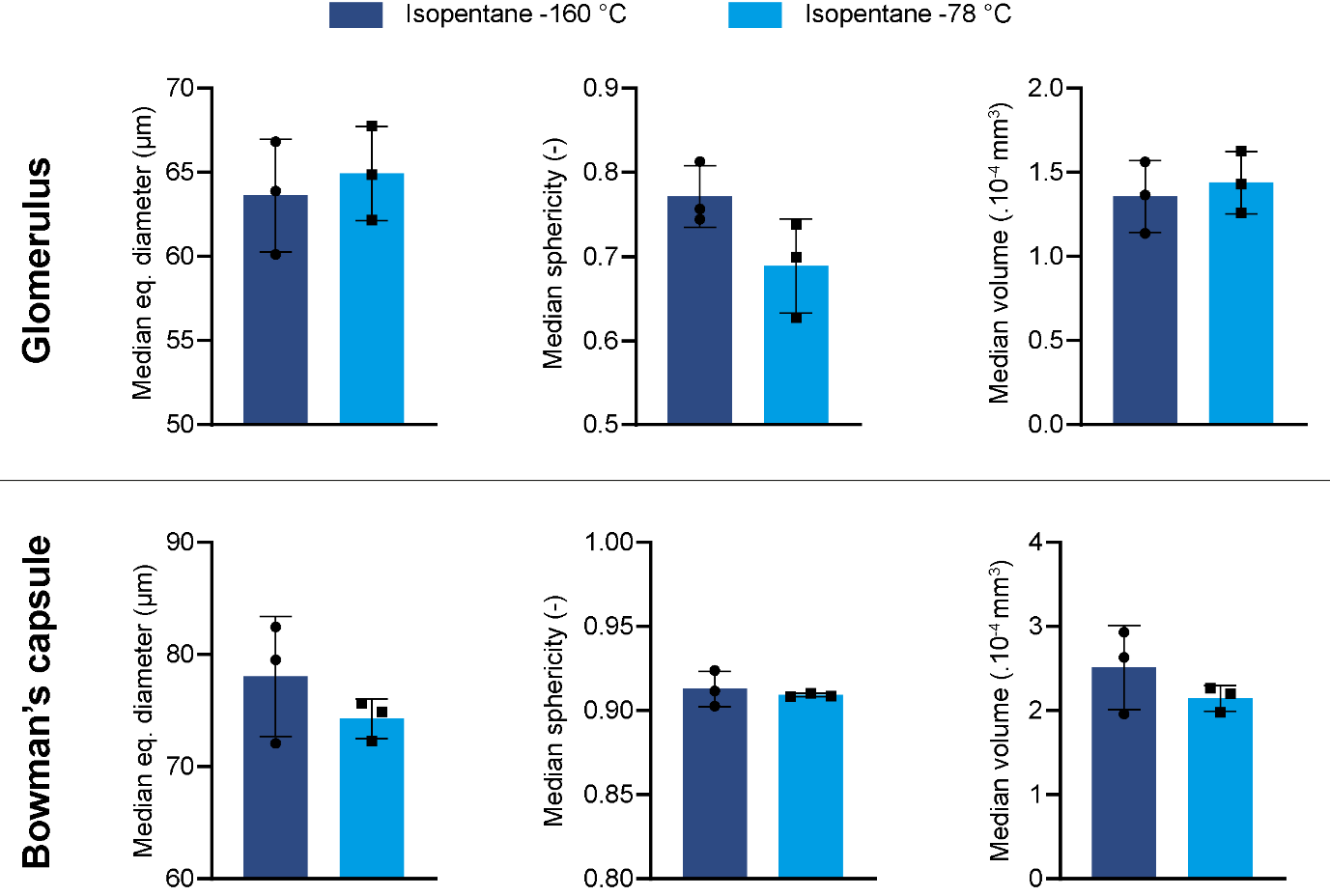


**Supplementary Fig. 6| Quantitative results of the structural characterization of the glomeruli and Bowman’s capsules.** Bar graphs showing the comparison of the freezing protocols isopentane -160 °C and isopentane -78 °C. Both for the glomeruli and Bowman’s capsules, results are shown for the median equivalent diameter, median sphericity and median volume. Individual data points (n = 3 for each condition) are indicated on the bar graphs. Two-sided unpaired t-testing resulted in no significant differences between the two freezing protocols (p > 0.05).


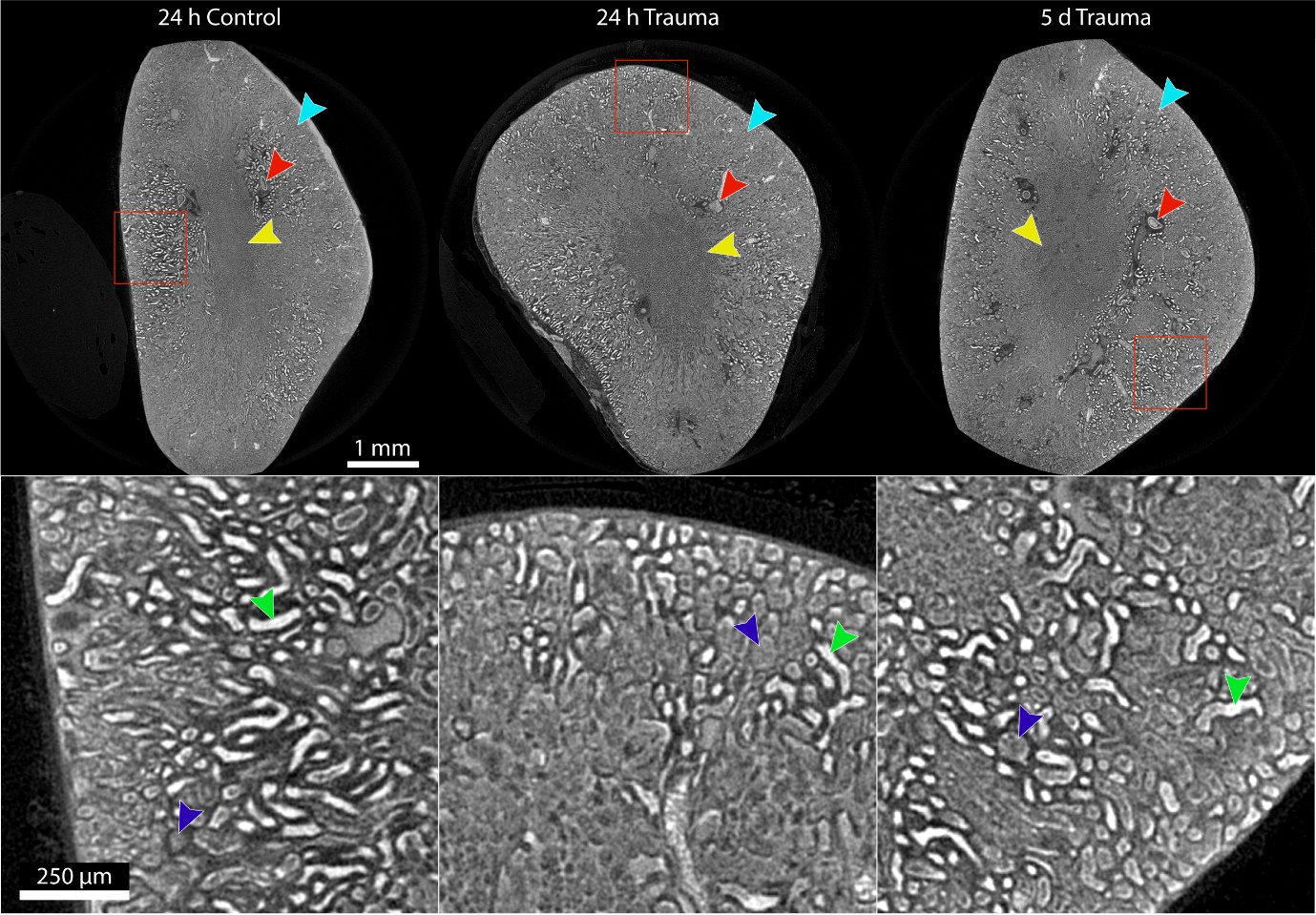


**Supplementary Fig. 7| Representative cryo-CECT slices for control and trauma groups in the TRAKI model.** Transverse cryo-CECT slices through the upper pole of the kidneys stained with Hf-WD POM and frozen using isopentane at -78 °C. The medulla (yellow arrow), cortex (light blue arrow) and blood vessels (red arrow) are indicated. Magnifications of the cortical region (red rectangle) are shown below, with the cortical tubuli (green arrow) and glomeruli (dark blue arrow) indicated.


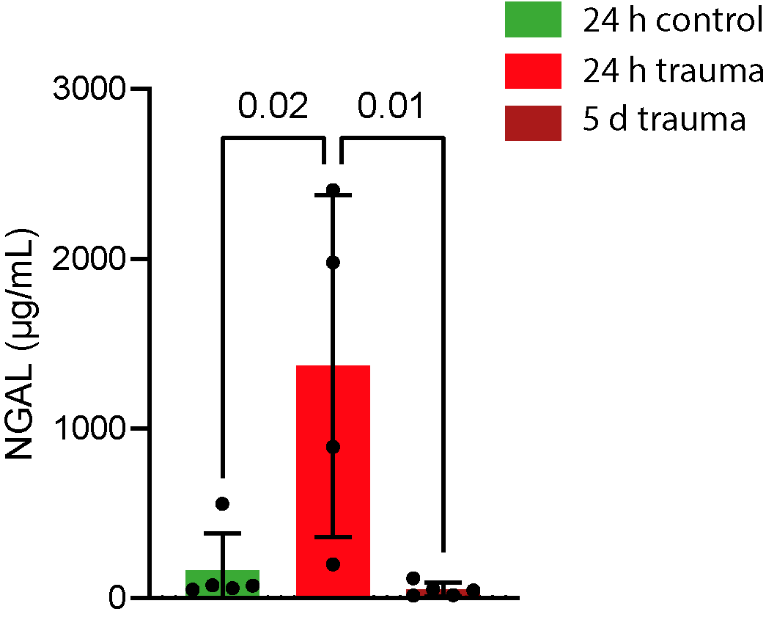


**Supplementary Fig. 8| NGAL in urine after trauma.** One-way analysis of variance, followed by a two-sided Tukey’s test, was conducted (n = 5 for 24 h control and 5 d trauma; and n = 4 for 24 h trauma). Significant p-values are indicated above the graphs (p < 0.05).


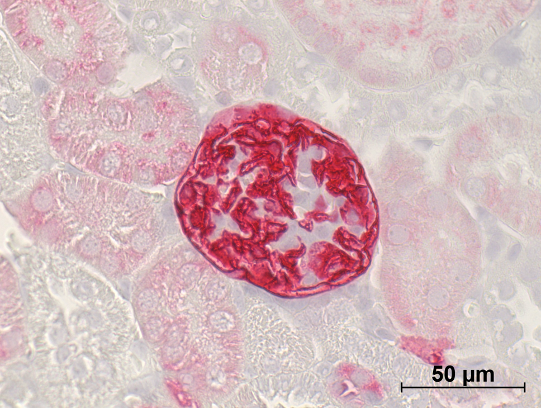

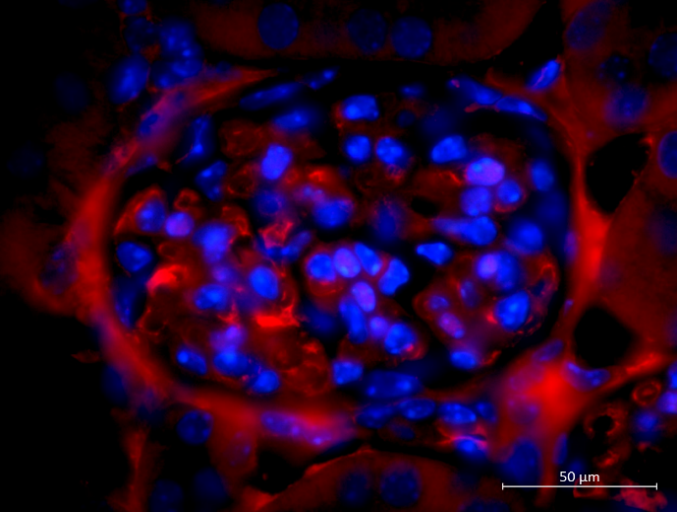


**Supplementary Fig. 9| Immunohistochemistry performed on kidneys that underwent cryo-CECT imaging (Hf-WD POM staining and freezing using isopentane at – 78 °C).** Visualization of the glomerulus after immunohistochemical staining with an antibody to podoplanin (podoplanin in red, nuclei in blue) and immunofluorescence staining with an antibody to GATA3 (GATA3 in red, nuclei in blue).


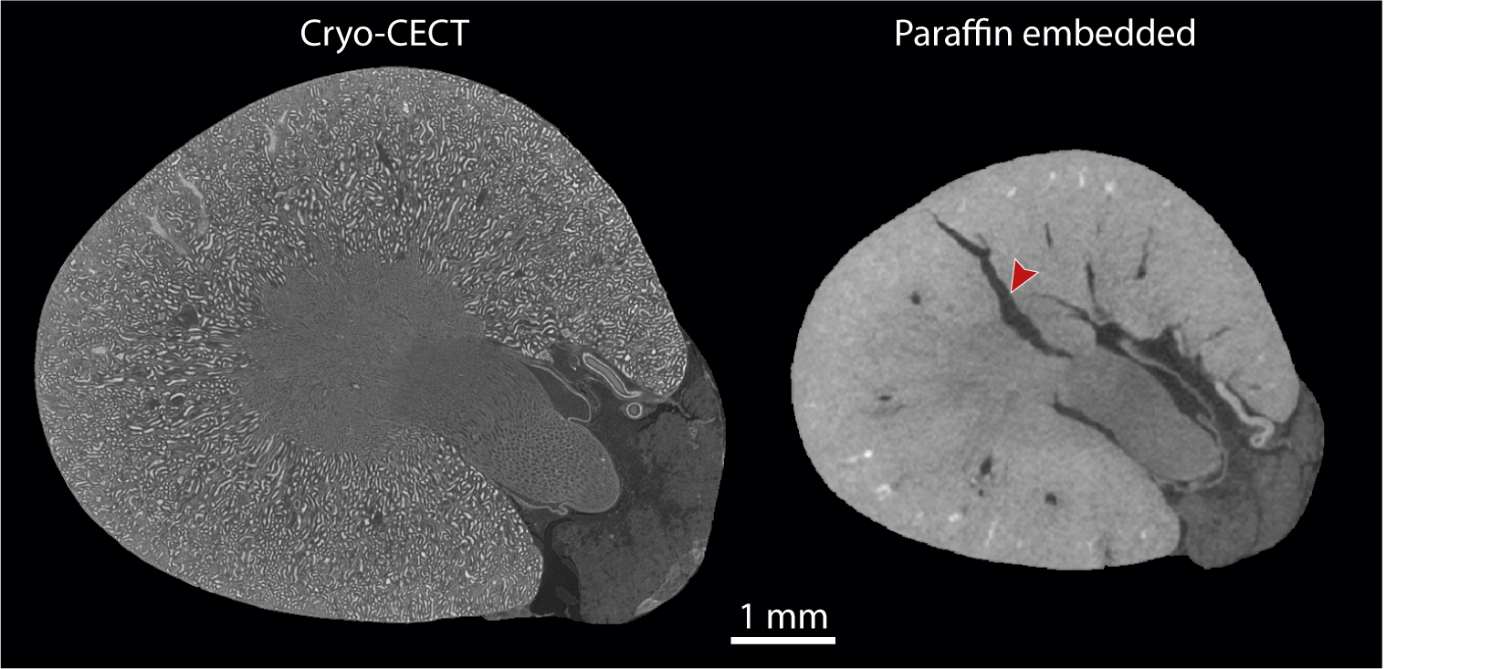


**Supplementary Fig. 10| The tissue shrinkage induced by dehydration and paraffin embedding as preparation for classical 2D histology.** Registered transverse slices of the same kidney using cryo-CECT (left) and embedded in paraffin (middle), showing the substantial size reduction of the paraffin embedded tissue (relative area reduction of 43%). The red arrow indicates a large crack in the paraffin embedded kidney.

**Supplementary Video 1 | 3D rendering of the kidney’s vasculature and two individual nephrons.** 3D rendering based on the cryo-CECT cortical zoom dataset, showing the renal artery (red) and two individual nephrons (blue) with their glomeruli (green). The branched artery eventually connects to the glomerulus of the nephron.

**Supplementary Video 2 | 3D rendering of several nephrons.** 3D rendering based on the cryo-CECT cortical zoom dataset, showing several individual nephrons, each being rendered in a different color. Glomeruli of the nephrons are rendered in red. Nephron segmentation was discontinued at the onset of the thin descending limb.
